# Pharmaco-invasive therapy: Early implementation of statins and proprotein convertase subtilisin/kexin type 9 inhibitors after acute coronary syndrome

**DOI:** 10.3389/fcvm.2022.1061346

**Published:** 2022-12-08

**Authors:** F. B. Mensink, J. Los, T. J. F. Ten Cate, R. M. Oemrawsingh, M. A. Brouwer, S. El Messaoudi, N. van Royen, J. H. Cornel, N. P. Riksen, R. J. M. van Geuns

**Affiliations:** ^1^Department of Cardiology, Radboud University Medical Center, Nijmegen, Netherlands; ^2^Department of Cardiology, Albert Schweitzer Ziekenhuis, Dordrecht, Netherlands; ^3^Department of Cardiology, Noordwest Ziekenhuisgroep, Alkmaar, Netherlands

**Keywords:** ACS, PCSK9 inhibition therapy, MACE, statins, LDL-C

## Abstract

Elevated LDL-cholesterol (LDL-C) plays a major role in atheroma formation and inflammation. Medical therapy to lower elevated LDL-C is the cornerstone for reducing the progression of atherosclerotic cardiovascular disease. Statin therapy, and more recently, other drugs such as proprotein convertase subtilisin/kexin type 9 (PCSK9) inhibitors, have proven efficacy in long-term lowering of LDL-C and therefore diminish cardiovascular risk. During an acute coronary syndrome (ACS), a systemic inflammatory response can destabilize other non-culprit atherosclerotic plaques. Patients with these vulnerable plaques are at high risk of experiencing recurrent cardiovascular events in the first few years post-ACS. Initiating intensive LDL-C lowering therapy in these patients with statins or PCSK9 inhibitors can be beneficial *via* several pathways. High-intensity statin therapy can reduce inflammation by directly lowering LDL-C, but also through its pleiotropic effects. PCSK9 inhibitors can directly lower LDL-C to recommended guideline thresholds, and could have additional effects on inflammation and plaque stability. We discuss the potential role of early implementation of statins combined with PCSK9 inhibitors to influence these cascades and to mediate the associated cardiovascular risk, over and above the well-known long-term beneficial effects of chronic LDL-C lowering.

## Introduction

Coronary artery disease (CAD) is a major cause of morbidity and mortality globally (1, 2). An elevated low-density lipoprotein cholesterol (LDL-C) level is strongly related to the development of CAD. Lowering LDL-C, either by lifestyle modification or by medication such as statins, results in long-term reduction of cardiovascular events, both in primary and in secondary prevention (3–6). The addition of the novel proprotein convertase subtilisin/kexin type 9 (PCSK9) inhibitors can drive LDL-C to even lower levels, further reducing the cardiovascular event rate in these patients (7). ACS patients have a very high risk of recurrent events (8), presumably due to an inflammatory state that can destabilize other non-culprit plaques (9). A rapid reduction of both LDL-C and inflammation is paramount in these high-risk patients. Furthermore, statins have anti-inflammatory properties as well, which makes them valuable in patients with ACS (10). Similarly, PCSK9 inhibitors are thought to have anti-inflammatory properties, and could be an interesting additional therapy option directly post-ACS.

In this review we discuss how elevated serum LDL-C and inflammation cause recurrent cardiovascular events by negatively influencing non-culprit plaque size and composition in patients post-ACS. We discuss the potential of early implementation of statins combined with PCSK9 inhibitors post-ACS to influence this process, over and above the known long-term beneficial effects of chronic LDL-C lowering.

## Long-term effects of low-density lipoprotein cholesterol reduction in patients with stable coronary artery disease

Sixty years ago, it was observed that patients with myocardial infarction often had high serum LDL-C (11). The Framingham study was first to systematically demonstrate elevated serum total cholesterol to be an independent risk factor for developing CAD in a population, alongside the effect of elevated blood pressure, and gender (12). The Scandinavian Simvastatin Survival Study (4S) demonstrated that lowering LDL-C with simvastatin, a drug in the new 3-hydroxy-3-methyl-glutaryl-coenzyme A (HMG-CoA) reductase inhibitor class, led to a large risk reduction in cardiovascular morbidity and mortality in high-risk patients with stable CAD. The Kaplan-Meier curve of the simvastatin arm diverged from the placebo arm only after one year of treatment (the median follow-up duration of this study was 5.4 years) (13). The 4S was subsequently followed by a multitude of outcome trials using various statins with different baseline levels of serum total cholesterol and LDL-C. The Cholesterol and Recurrent Events (CARE) (median follow-up: 5.0 years) and Long-term Intervention with Pravastatin in Ischaemic Disease (LIPID) (median follow-up: 6.1 years) trials show that statin treatment improves cardiovascular outcomes for patients with CAD, regardless of baseline serum cholesterol levels. Relative risk reductions vary from 24 to 30% after 5 to 6 years of follow-up. In these trials, the Kaplan-Meier curves diverged after 1–2 years of treatment as well, solidifying the evidence that the effects of statins in stable CAD patients emerge relatively late (14, 15). An overview of landmark LDL-C lowering trials can be found in the Supplementary material.

**FIGURE 1:**
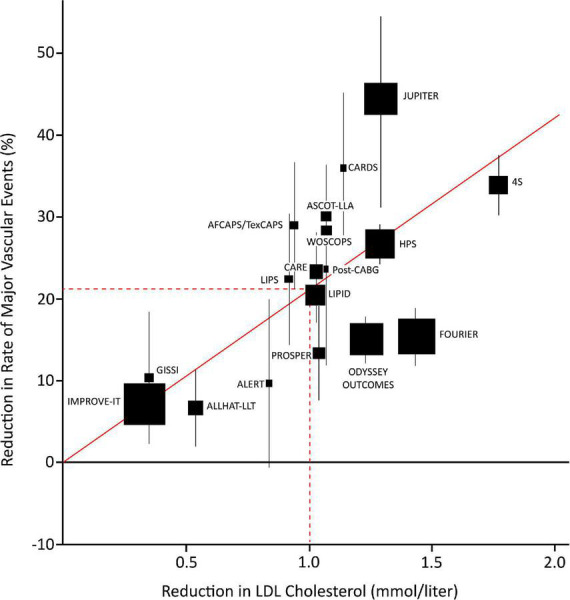
Plot of statin, ezetimibe, and PCSK9 trials depicting the relationship between mean LDL-cholesterol (LDL-C) reduction and the proportional reduction of major vascular events (MVE) at one year (MVE: composite of death from coronary heart disease, myocardial infarction, stroke, or revascularization more than 30 days after randomization). Squares represent single trials, the size of the squares represent the number of endpoints in the study. The vertical lines represent one standard error (SE). The regression line represents the event rate reduction per mmol/l LDL-C reduction. AFCAPS/TexCAPS, Air Force/Texas Coronary Atherosclerosis Prevention Study; ALERT, Assessment of Lescol in Renal Transplantation; ALLHAT-LLT, Antihypertensive and Lipid-Lowering Treatment to Prevent Heart Attack Trial–Lipid Lowering Trial; ASCOT-LLA, Anglo-Scandinavian Cardiac Outcomes Trial–Lipid Lowering Arm; CARDS, Collaborative Atorvastatin Diabetes Study; CARE, Cholesterol and Recurrent Events; FOURIER, Further Cardiovascular Outcomes Research With PCSK9 Inhibition in Subjects With Elevated Risk; GISSI, Gruppo Italiano per lo Studio della Sopravvivenza nell’Infarto Miocardico; HPS, Heart Protection Study; IMPROVE-IT, Improved Reduction of Outcomes: Vytorin Efficacy International Trial; JUPITER, Justification for the Use of Statin in Prevention: An Intervention Trial Evaluating Rosuvastatin; LDL, low density lipoprotein; LIPID, Long-term Intervention with Pravastatin in Ischemic Disease; LIPS, Lescol Intervention Prevention Study; MVE, major vascular events; ODYSSEY, Evaluation of Cardiovascular Outcomes After an Acute Coronary Syndrome During Treatment With Alirocumab; Post CABG, Post–Coronary Artery Bypass Graft; PROSPER, Long-term Intervention with Pravastatin in Ischemic Disease; SE, standard error; WOSCOPS, West of Scotland Coronary Prevention Study; 4S, Scandinavian Simvastatin Survival Study. Figure edited from Cannon et al. (98), with permission.

Today, statins are the cornerstone in the pharmacological management of elevated serum LDL-C levels in patients at high risk for cardiovascular events (10). A review that compiled results from several contemporary randomized clinical trials (RCTs), meta-analyses on LDL-C reduction, and cardiovascular outcomes, reported a 22% proportional reduction in the rate of major adverse cardiovascular events (MACE) for every 1 mmol/L serum LDL-C reduction with statin therapy every year (MACE defined as coronary death, MI, stroke, and coronary revascularization) (16). This relationship between LDL-C and major vascular events is presented in Figure 1. Recent evidence suggests that similar relationships can be found between MACE and the reduction of other lipoproteins, like ApoB. A systematic review by Michos et al. (17) compiled the results of 29 trials reporting ApoB lowering and found a 5% relative reduction of MACE for every 10 mg/dl reduction in ApoB.

## Early effects on cardiovascular events in patients post-acute coronary syndrome

Risk reduction is particularly important in patients at high risk for recurrent ischemic events, especially those who suffered an acute coronary syndrome (ACS) (10). The majority of the initial statin studies excluded patients with recent acute myocardial infarction (AMI) or unstable angina pectoris (UAP), a point of contention as the majority of cardiovascular events occur in the early phase post-ACS. A large Swedish national registry study of 108,315 patients hospitalized with MI showed that 18.3% experienced a recurrent event in the first 365 days after the index event. The risk for a recurrent event in the following 3 years was 20% (8). In these high-risk or “vulnerable” patients, early, rapid, and intense LDL-C reduction may reduce the risk of recurrent events. This has been investigated in a limited number of studies: the myocardial ischemia reduction with aggressive cholesterol lowering (MIRACL) (high-intensity atorvastatin versus placebo) (18), the pravastatin or atorvastatin evaluation and infection therapy–thrombolysis in myocardial infarction 22 (PROVE IT-TIMI 22) (high-intensity atorvastatin versus medium-dose pravastatin) (19), and statins evaluation in coronary procedures and revascularization (SECURE-PCI) (20) trials. The MIRACL trial found a significant 2.6% absolute risk reduction, and 16% relative risk reduction in MACE after a treatment period of 16 weeks (MACE defined as death, non-fatal acute MI, resuscitated cardiac arrest, or recurrent symptomatic myocardial ischemia with objective evidence and emergency rehospitalization) (18). In a sub-analysis of the PROVE IT-TIMI 22, a significant 28% relative risk reduction in MACE was found after just 30 days of treatment (3% event rate in the intensive statin therapy group vs. 4.2% in the standard statin therapy group; MACE defined as death, MI, and rehospitalization). This effect was seen as early as 15 days after start of treatment (21). The SECURE-PCI trial determined the effect of periprocedural dosing of high-intensity statin therapy on MACE after 30 days. Although there was no significant reduction in MACE after 30 days in the overall ACS population, a significant effect in the subgroup of patients who underwent PCI was found (MACE defined as all-cause mortality, MI, stroke, and unplanned coronary revascularization) (20). These RCTs suggested that starting high-intensity statin therapy could improve early cardiovascular outcomes, when implemented directly post-ACS. These trials (see Table 1 for an overview) formed the basis for the current implementation of high-intensity statin therapy as standard-of-care approach in patients with ACS. Despite this approach, a substantial residual risk of recurrent MACE remains (10).

**TABLE 1:** Trials with early initiation of low density lipoprotein-cholesterol (LDL-C) lowering therapy post-acute coronary syndrome (ACS).

Study	Population	Treatment	Primary endpoint	Results	Median FU
SECURE-PCI	ACS *n* = 4191	Atorvastatin 80 mg loading dose 24 h before and after PCI vs. placebo 40 mg atorvastatin for 30 days after second dose of study medication (all patients)	MACE (all-cause mortality, MI, stroke, unplanned coronary revascularization)	HR 0.88 (95% CI: 0.69–1.11; *p* = 0.27)	30 days
MIRACL	AMI and UAP *n* = 3086	Atorvastatin 80 mg vs. placebo	MACE (death, non-fatal MI, cardiac arrest with resuscitation, recurrent symptomatic myocardial ischemia)	RR 0.84 (95% CI: 0.70–1.00; *p* = 0.048) RR 0.74 for recurrent symptomatic ischemia (95% CI: 0.57–0.95; *p* = 0.02)	16 weeks
PROVE IT-TIMI 22	ACS *n* = 4162	Pravastatin 40 mg vs. Atorvastatin 80 mg	MACE (death from any cause, MI, UAP requiring hospitalization, revascularization, stroke)	HRR 16% (95% CI: 5–26%; *p* = 0.005)	2.0 years (effect apparent after 30 days)

Landmark trials on the effect of early initiation of low density lipoprotein-cholesterol (LDL-C) lowering therapy post-ACS on cardiovascular endpoints. Table is organized by follow-up time. ACS, acute coronary syndrome; FU, follow-up; HR, hazard ratio; HRR, hazard ratio reduction; MACE, major adverse cardiac events; MI, myocardial infarction; RR, relative risk; UAP, unstable angina pectoris.

## The inflammation hypothesis

Patients with UAP or recent AMI experience an early positive effect from statin therapy, while patients with stable CAD only experience benefit from statin therapy after multiple years. This slow and long-term beneficial effect on cardiovascular events is largely related to the long-term drop in serum LDL-C levels (10). These fat soluble lipoprotein particles can easily migrate through the endothelium. So when the concentration of circulating LDL-C is too high, LDL-C accumulates in the intima, initiating atheroma formation (22). Lowering LDL-C with high-intensity statin therapy can halt and even partially reverse this process (23–25).

However, this happens gradually and therefore does not explain the very early benefits from high-intensity statin therapy observed in patients with unstable CAD. Another phenomenon can be linked to the early cardiovascular event rate is inflammation. The anti-inflammatory effects of statins may be the basis for the early benefit on cardiovascular events.

C-reactive protein (CRP) is an acute-phase protein that is released as a response to inflammation. High-sensitive CRP (hsCRP) assays can detect very low concentrations of CRP. HsCRP is therefore the marker most commonly used in contemporary studies that aim to link the inflammatory process to atherosclerosis. Burke et al. (26) found that some patients who died from sudden coronary death due to severe CAD had also elevated levels of CRP. CRP was subsequently identified as an independent predictor for cardiovascular events (9). *Post-hoc* analyses of the AFCAPS/TexCAPS and CARE found that cardiovascular risk was increased in patients with higher baseline levels of CRP. This increased risk was attenuated in patients who received statin therapy (27–30). The first real prospective and randomized study that investigated the effect of statin therapy on CRP levels was the PRINCE study, which demonstrated that statin therapy indeed lowered CRP (31). Successively, Nissen et al. (32) found that the reduced progression of CAD through high-intensity statin therapy, as measured by intravascular ultrasound (IVUS), was not only significantly related to reduction in LDL-C, but also to reduction in CRP. Lastly, Ridker et al. (33, 34) found that patients who had lower CRP after treatment with statins had lower residual cardiovascular risk than patients with higher CRP levels. These findings confirm that statin therapy has an anti-inflammatory effect, and that the reduction of CRP by statins is accompanied by a beneficial effect on clinical outcomes.

Moreover, post-mortem histological analysis of ruptured plaques in patients with AMI revealed that these plaques were often heavily infiltrated by leukocytes, which also suggests there is a link between active inflammation and unstable CAD (34). Joshi et al. (35) used combined positron emission and computed tomography to measure aortic fludeoxyglucose (FDG)-uptake in 40 patients with AMI and 40 patients with stable angina. Patients with AMI had a higher aortic FDG uptake than patients with stable angina, despite having similar aortic atherosclerotic burden. This reflects an increased plaque inflammation in patients with AMI (35). It is thought that plaque inflammation can lead to plaques that are more prone to rupture or erosion, and can therefore cause an ACS, leading to severe morbidity and mortality (36, 37). The pathologist Virmani found that patients who had died from AMI frequently possessed inflamed plaques with distinctive morphological characteristics (38). These plaques contained a large lipid pool with a necrotic core, covered by a thin fibrotic cap (39). During plaque growth, the different cells such as lipid-laden foam cells and smooth muscle cells (SMCs) inside the plaque are deprived of oxygen and undergo apoptosis, resulting in a necrotic core (40). Covering the necrotic core is a thin layer of protective collagen tissue (often <65 microns), separating the blood in the coronary artery from the thrombogenic contents of the plaque underneath. Virmani gave this type of plaque the descriptive term “thin-cap fibroatheroma” (TCFA). In recent pathological and optical coherence tomography (OCT) imaging studies, patients with AMI and ACS often had other TCFA lesions in the coronary tree, emphasizing the high-risk profile of these patients (41–44).

## Influencing inflammation by direct low-density lipoprotein cholesterol reduction

Elevated serum LDL-C can directly influence inflammation *via* the formation of oxidized LDL (OxLDL). A recent article by Jukema et al. (45) describes that minimally modified LDL-C (MM-LDL) can influence inflammation by activating the innate immune system *via* toll-like receptors (TLRs). Minimally modified low-density lipoprotein is an LDL particle that contains oxidized lipid molecules (OxPC) that is also recognized by the LDL receptor. TLRs can in turn activate the NLR family pyrin domain containing 3 (NLRP3) inflammasome which leads to the production of pro-inflammatory cytokines. NLPR3 promotes inflammation through the IL-1bèta and IL-18 pathway. OxLDL is created when lipid molecules in the LDL particle become oxidized, in addition to the LDL surface protein beta (ApoB) (46). As a result the OxLDL is no longer recognized by the LDL receptor anymore, but instead binds to scavenger receptors on macrophages, promoting foam cell formation and inflammation (47).

Recent experimental evidence has also shown that the occurrence of AMI temporarily accelerates atherogenesis throughout the body. In ApoE-/- mice, coronary ligation rapidly increased lesion size, inflammatory macrophage content, and protease activity in aortic atherosclerotic plaques as early as 1 week post-AMI, which persisted for at least 12 weeks (48). This acceleration was caused by increased proliferation and activation of myeloid cell progenitors in the bone marrow (49). One mechanism that might contribute to this persistent immune system activation post-AMI is trained immunity, which is defined as the development of long-term hyperresponsive innate immune cells following brief stimulation of these cells (50). Although this mechanism is beneficial in the context of recurrent infections, it can be detrimental in situations of chronic low-grade inflammation, such as atherosclerosis. This trained immune phenotype develops due to metabolic and epigenetic reprogramming of myeloid cell progenitors and can persist for weeks (51). Several endogenous atherogenic compounds can induce trained immunity, including OxLDL (52), lipoprotein (a) (53), and the catecholamines noradrenaline and adrenaline (54), which temporarily increase after an AMI.

Given the role of oxidized lipoproteins as triggers of inflammation and trained immunity, and the role of intracellular cholesterol in proliferation of myeloid progenitor cells in the bone marrow (55), lowering cholesterol immediately post-AMI could reduce the inflammatory response and trained immunity induction.

## Benefits beyond direct low-density lipoprotein cholesterol reduction

Influencing inflammation by direct LDL-C reduction is not the only means by which statins could exert early clinical benefits after ACS. These benefits are thought to be attributable to the effects of statins in several other fields as well. These so-called pleiotropic effects include additional anti-inflammatory properties that are independent from LDL-C lowering, and positive effects on endothelial function and coagulation, both of which are dysregulated directly after an ACS occurs (21, 56). This could explain how early statin therapy post-ACS can affect the cardiovascular event rate.

The possible detrimental role of an ACS on endothelial function and coagulation and its attenuation by statins are thoroughly discussed by Ray et al. (56). The reduction of cholesterol in ischemia and function of the endothelium (RECIFE) trial found a 42% improvement in endothelial function after 6 weeks of statin therapy compared to placebo in patients with ACS (57). In another study, high levels of circulating adhesion molecules were associated with higher cardiovascular risk (58), and in successive studies these circulating adhesion molecules were reduced by statin therapy (59, 60). Secondly, statins possess various antithrombotic properties. Statins can decrease prothrombotic tissue factor (TF) expression on the endothelium, reduce thrombin generation, and influence several other pro-coagulant cascades (61). In addition, statins can prevent the development of aforementioned trained immunity in monocytes and macrophages by preventing accumulation of intracellular mevalonate (52, 62).

The discovery of pleiotropy in statins triggered the design of multiple outcome trials. Some trials focused on inflammation and the immune modulation of statins in particular. One such trial, the JUPITER, investigated the effect of statin therapy on MACE in apparently healthy individuals with elevated CRP, but without elevated LDL-C levels. Rosuvastatin 20 mg or placebo was administered daily, with a median follow-up duration of 1.9 years. There was a significant reduction of cardiovascular events in the group that received rosuvastatin. This risk reduction was far greater than what was expected from LDL-C reduction alone (63). Research on pleiotropic effects of statins clearly shows that there is a myriad of possible mechanisms by which statins can influence inflammation, endothelial function, and coagulation, possibly explaining the effect on cardiovascular outcome.

## Proprotein convertase subtilisin/kexin type 9 inhibitors: Long-term effect on cardiovascular events in patients with stable coronary artery disease

The potentially pivotal role of PCSK9 in LDL-C regulation was discovered in the early 21th century by the efforts of different study groups. Researchers discovered that gain-of-function mutations in the PCSK9 gene cause hypercholesterolemia and that loss-of-function mutations in the PCSK9 gene resulted in the opposite (64–66). These loss-of-function mutations were subsequently associated with a lower incidence of CAD (67). The underlying mechanism of action of the PCSK9 enzyme was elucidated, which in turn paved the way for possible therapeutic targets (68–70). Specific monoclonal antibodies that bind and inactivate the PCSK9 enzyme were developed and tested for efficacy and safety (71, 72). Large clinical trials have been performed to elucidate the effects of PCSK9 inhibitors evolocumab (73) (FOURIER) and alirocumab (ODYSSEY OUTCOMES) (74) on long-term cardiovascular events. In these studies, patients treated with PCSK9 inhibitors reached the lower LDL-C targets more often and a significant reduction in cardiovascular events was found. In the FOURIER, the primary composite endpoint occurred in 1,344 patients (9.8%) in the evolocumab group and in 1,563 patients (11.3%) in the placebo group after a median follow-up of 2.2 years (HR 0.85; 95% CI: 0.79–0.92; *p* < 0.001) (composite endpoint defined as cardiovascular death, myocardial infarction, stroke, hospitalization for unstable angina, or coronary revascularization) (73). The ODYSSEY OUTCOMES reported similar results with the composite primary endpoint event occurring in 903 patients (9.5%) in the alirocumab group and in 1,052 patients (11.1%) in the placebo group after a median follow-up of 2.8 years (HR 0.85; 95% CI: 0.78–0.93; *p* < 0.001) (composite endpoint defined as death from CHD, non-fatal MI, fatal or non-fatal ischemic stroke, or unstable angina requiring hospitalization) (74). This reduction in cardiovascular events can be largely explained by the direct beneficial effect of more profound LDL-C lowering.

Imaging studies that add PCSK9 inhibitors to background statin therapy and investigate the effect of that combined therapy on plaque size have been performed, akin to the prior IVUS and Near Infrared Spectroscopy (NIRS) studies with statins only. The GLAGOV trial randomized patients with angiographically identified CAD to either statin therapy and placebo, or to statin therapy and evolocumab. Baseline IVUS images of atherosclerotic plaques were compared with follow-up images (follow-up duration 78 weeks), and the percent change in atheroma volume (PAV) and total change in atheroma volume (TAV Both PAV and TAV decreased significantly in the evolocumab group (75). These studies demonstrate that PCSK9 inhibitors added to statin therapy can have a beneficial effect on plaque volume in patients with stable CAD.

## Proprotein convertase subtilisin/kexin type 9 inhibitors: Early effects on cardiovascular events in patients post-acute coronary syndrome?

The FOURIER and ODYSSEY OUTCOMES demonstrated the benefit of PCSK9 monoclonal antibodies on cardiovascular events in patients with stable CAD, which is primarily driven by more profound LDL-C lowering. PCSK9 inhibitors might have a beneficial effect on cardiovascular events directly post-ACS, by targeting inflammation. There is experimental evidence of ways by which PCSK9 antibodies could exert effects directly post-ACS, analogous to the pleiotropic effects of statins.

In a study of male rats, plasma PCSK9 levels were markedly elevated from 12 to 96 h after they suffered from AMI (76). In humans, plasma PCSK9 levels seem to be higher in patients with an inflammatory response directly post-ACS, and higher plasma PCSK9 can in turn promote inflammation, which heightens the risk for recurrent ischemic events (77, 78). Thus, PCSK9 inhibitors administered directly post-ACS could break this positive feedback loop of inflammation (79).

**FIGURE 2:**
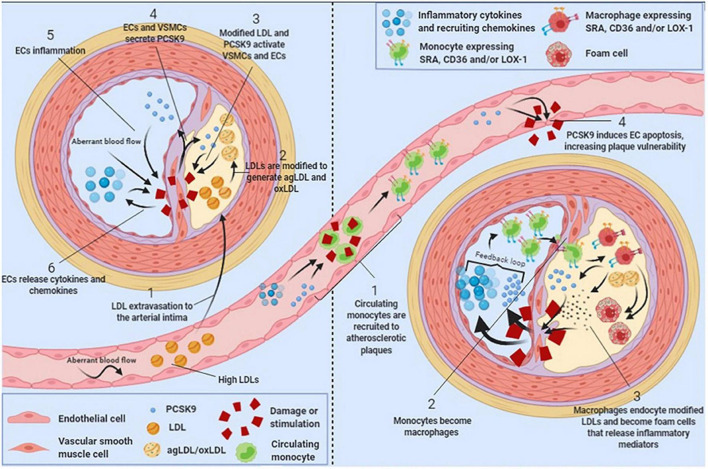
Schematic showing the role of local intraplaque proprotein convertase subtilisin/kexin type 9 (PCSK9) in atherosclerosis progression. Figure reprinted from Luquero et al. (99) with permission from the authors.

**FIGURE 3:**
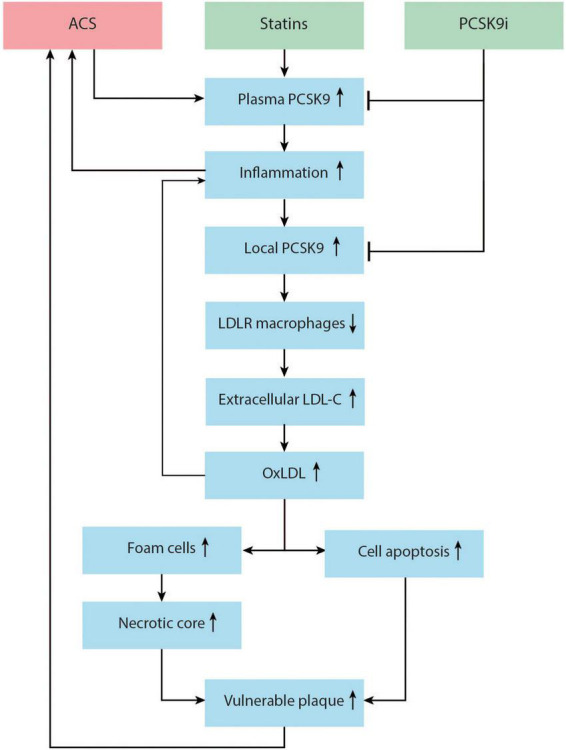
Proposed mechanism of the role of proprotein convertase subtilisin/kexin type 9 (PCSK9) enzyme in the promotion of vulnerable plaque and recurrent acute coronary syndrome (ACS). ACS and statin therapy can both upregulate plasma PCSK9. Plasma PCSK9 can promote inflammation, increasing the risk for recurrent ACS. Local inflammation in the vessel wall can upregulate local PCSK9 production by smooth muscle cells (SMCs). Local PCSK9 can downregulate macrophage low density lipoprotein-cholesterol (LDL-C) receptors, decreasing LDL-C uptake and increasing the pool of extracellular lipid. This extracellular LDL-C is transformed to oxLDL, which activates pro-inflammatory genes and pro-inflammatory cytokine production, and induces SMC and endothelial cell apoptosis. Macrophages take up oxLDL *via* scavenger receptors, thus promoting foam cell formation and necrotic core formation. This process might increase vulnerable plaque and risk for recurrent ACS. PCSK9 inhibitors lower plasma PCSK9 and might also lower local PCSK9 in the vessel wall, inhibiting this process.

The inflammatory response post-ACS is only partially negated by the early implementation of high-intensity statin therapy, which modulates inflammation and an halt the progression of atherosclerotic plaques (25, 63). However, in reaction to high-intensity statin therapy, PCSK9 enzyme can be upregulated in an effort to keep equilibrium. This negative feedback mechanism has been observed in humans treated with high-dose atorvastatin (80). The PCSK9-REACT study found that patients with increased concentrations of plasma PCSK9 also had a significantly higher platelet activity, indicating yet another possibility by which elevated plasma PCSK9 levels can increase the risk of future cardiovascular events (81). Adding PCSK9 inhibitors to this high-intensity statin regimen directly post-ACS may mitigate these reactions. It is not yet clear if PCSK9 inhibitors will have a noticeable effect on inflammation *in vivo*. A meta-analysis on PCSK9 inhibitors and CRP levels did not find a significant change in CRP after PCSK9 inhibitor therapy. However, the patients in these RCTs had low baseline CRP levels and did not have unstable coronary artery disease (82). The EVOCATION trial investigated whether administering PCSK9 inhibitors 6 weeks prior to PCI could decrease periprocedural microvascular resistance due to possible anti-inflammatory properties, but no significant effect was found (83). Conversely, significant effects might be found in patients with recent ACS, or with longer duration of PCSK9 inhibitor treatment. Interestingly, a recent study by Hoogeveen et al. (84) found a decreased arterial wall inflammation in fifty patients with established CAD, imaged by PET/CT, after 14 weeks of alirocumab treatment, while systemic inflammatory markers did not change. It could therefore be hypothesized that PCSK9 inhibitors can influence inflammation more locally in the vessel wall. Early *in vitro* and animal studies suggest that PCSK9 enzyme can be produced locally in the atherosclerotic plaque by smooth muscle cells and can signal in a paracrine manner (85) (Figure 2). In these studies, PCSK9 downregulated intraplaque macrophage surface LDL-C receptors. It is theorized that this leads to impaired uptake and efflux of LDL-C by macrophages and to the accumulation of extracellular lipid (79, 86, 87). Nearby macrophages can transform extracellular LDL-C into OxLDL-C (86). OxLDL-C has multiple pro-inflammatory effects. It can activate pro-inflammatory genes of endothelial cells, smooth muscle cells, and macrophages (88). As mentioned earlier, OxLDL-C can be phagocytized by macrophages *via* scavenger receptors, promoting foam cell formation. New evidence suggest that some scavenger receptors then upregulate PCSK9 expression (87, 89). The presence of OxLDL-C can lead to apoptosis of smooth muscle cells and endothelial cells, promoting endothelial permeability and fibrous cap thinning (90). The inhibition of PCSK9 might attenuate all these negative effects of OxLDL-C (79, 87, 90, 91). The flowchart in Figure 3 demonstrates a proposed mechanism by which PCKS9 could directly influence plaque vulnerability.

Kühnast et al. (92) demonstrated that PCSK9 inhibitor alirocumab improved plaque size and composition in hypercholesterolemic mice. Alirocumab decreased aortic atherosclerotic plaque size, lesion macrophage area, and necrotic core area. Conversely, alirocumab increased fibrous cap thickness, and collagen area. It is not clearly established if these effects of local PCSK9 are clinically relevant in humans. However, the ATHEROREMO-IVUS study found a linear relationship with the serum levels of PCSK9 enzyme in patients with stable CAD and the necrotic core volume and necrotic core percentage of the atherosclerotic plaque. This association was independent of LDL-C levels, suggesting that PCSK9 is directly influencing plaque composition, presumably *via* the pathways described above (93). All this preliminary evidence on the detrimental role of PCSK9 enzyme in the post-ACS setting warrant more research on the use of PCSK9 inhibitors directly after an ACS occurs.

## Contemporary and future studies with proprotein convertase subtilisin/kexin type 9 inhibitors

The EVOPACS study proved that adding the PCSK9 inhibitor evolocumab directly post-ACS is safe and can effectively lower LDL-C levels to below guideline recommendations in >95% of patients after 8 weeks (94). The EVACS study recently demonstrated that the early initiation of PCSK9 inhibitors post-ACS rapidly and significantly reduces LDL-C levels in just 24 h, and that AHA/ACC and ESC guideline LDL-C levels for secondary prevention can be achieved at hospital discharge for most patients (95). Imaging studies that investigate the effect of PCSK9 inhibitors administered directly post-ACS on plaque size and morphology have recently been published.

The PACMAN-AMI examined the effect of alirocumab administered directly post-ACS on mean change in PAV (primary endpoint) measured by IVUS, on lipid core burden index (LCBI) measured by NIRS, and on fibrous cap thickness (FCT) measured by OCT. After 52 weeks, mean PAV decreased by 2.13% with alirocumab vs. 0.92% with placebo (difference: 1.21%; 95% CI: 1.78–0.65%; *p* < 0.001). The mean maxLCBI_4*mm*_ decreased by 79.42 with alirocumab vs. 37.60 with placebo (difference: 41.24; 95% CI: 70.71–11.77; *p* = 0.006). Mean minimal FCT increased by 62.67 μm with alirocumab vs. 33.19 μm with placebo (difference: 29.65 μm; 95% CI: 11.75–47.55; *p* = 0.001). These results reflect a decrease in plaque size and amelioration of plaque composition (96). The HUYGENS trial investigated the effect of evolocumab on absolute change in minimum FCT and lipid arc, measured by OCT. At 50 weeks, the minimum FCT increased by 42.7 μm in the evolocumab group vs. 21.5 μm in the placebo group (*p* = 0.015). The maximum lipid arc decreased by 57.5° in the evolocumab group vs. 31.4° in the placebo group (*p* = 0.04). Similar to the PACMAN-AMI, the HUYGENS found a greater decrease in PAV with evolocumab compared to placebo (2.29 ± 0.47% vs. –0.61 ± 0.46%; *p* = 0.009) (97).

The FITTER (NCT04141579) will investigate the effect of administration of evolocumab directly post-ACS (first dose within 24 h of index angiography) on the fractional flow reserve (FFR) of non-culprit lesions and LCBI of the non-culprit vessel. During the index angiography, FFR of existing non-culprit lesions will be measured. Evolocumab will be administered for a duration of 3 months, after which follow-up FFR is performed. Change in FFR will be the primary endpoint of this study. Secondly, LCBI of the non-culprit vessel will be compared between baseline and follow-up.

All these outcome measures describe various morphological and physiological aspects of the atherosclerotic plaque. These properties might predict the vulnerability and the proneness of the plaque to cause future ischemia and ACS. However, these measures cannot substitute long-term studies with hard MACE endpoints. Thus, when PCSK9 inhibitors administered directly post-ACS has a beneficial effect on plaque size, morphology and physiology, more research with hard MACE endpoints is warranted.

## Conclusion

Statin therapy has proven efficacy in reducing the risk of recurrent events in patients with stable CAD and ACS. This risk reduction is primarily driven by profound long-term LDL-C reduction, although anti-inflammatory and other pleiotropic properties probably play an important role in the early stages post-ACS. Administering PCSK9 inhibitors directly post-ACS leads to an immediate and more pronounced LDL-reduction. Imaging studies that added PCSK9 inhibitors directly post-ACS demonstrated a stabilization of non-culprit plaques with an improvement of plaque composition, but information on the reduction of inflammation remains scare. The immediate addition of PCSK9 inhibitors to statin therapy has the potential to reduce recurrent events after ACS, but clinical endpoint studies are needed before this strategy can be adopted by the guidelines.

## Author contributions

FM wrote the manuscript. JL, TT, RO, MB, SE, NR, JC, and NPR helped in drafting the manuscript. RG was the main supervisor and helped in writing this review manuscript. All authors critically revised the review manuscript and approved the final version.
