# Supplementary material for: Pharmaco-invasive therapy: Early implementation of statins and proprotein convertase subtilisin/kexin type 9 inhibitors after acute coronary syndrome
Source: Front Cardiovasc Med. 2022 Dec 8;9:1061346. doi: 10.3389/fcvm.2022.1061346 (PMC9772027; doi:10.3389/fcvm.2022.1061346)
Supplement: Supplementary file 1 [file Table_1.PDF]

SUPPLEMENTARY MATERIAL

**Trials with late initiation of LDL-C lowering therapy in patients without CAD or with stable CAD**						
**Study**	**Population**	**Treatment**	**Time from index event to treatment**	**Primary endpoint**	**Results**	**Median FU**
AFCAPS/TexCAPS	No CAD<br>n= 6605	Lovastatin 20-40mg<br>vs. placebo	N/A	MACE (fatal/nonfatal MI, UAP, SCD)	RR 0.63<br>(95% CI: 0.50 to 0.79; p<0.001)	5.2 years
JUPITER	No CAD, high CRP<br>n = 17802	Rosuvastatin 20mg<br>vs. placebo	N/A	MACE (MI, stroke, arterial revascularization, hospitalization for UAP, cardiovascular death)	HR 0.56<br>(95% CI: 0.46 to 0.69; p<0.00001)	1.9 years
Cholesterol and Recurrent Events (CARE)	Stable CAD<br>n= 4159	Pravastatin 40mg<br>vs. placebo	10 ± 5 months<br>Mean ± SD	MACE (fatal coronary event, nonfatal MI)	RRR 24%<br>(95% CI: 9% to 36%; p=0.003)	5.0 years
Scandinavian simvastatin survival study (4S)	Stable CAD<br>n = 4444	Simvastatin 10- 40mg<br>vs. placebo	> 6 months	Total mortality	RR 0.70<br>(95% CI: 0.58 to 0.85; p=0.003)	5.4 years
MRC/BHF Heart Protection Study	Stable CAD<br>n = 20536	Simvastatin 40mg<br>vs. placebo	Not reported	MACE (mortality, fatal/nonfatal vascular events)	RR 0.87 for any death<br>(95% CI 0.81 to 0.94; p=0.0003)<br>RR 0.83 for any vascular<br>(95% CI: 0.75 to 0.91; p=<0.0001)	5.0 years
FOURIER	Stable CAD<br>n = 27564	Evolocumab 140mg<br>vs. placebo	3.4 (1.0 – 7.4)<br>Median (IQR)	MACE (cardiovascular death, MI, UAP requiring hospitalization, coronary revascularization, stroke)	HR 0.85<br>(95% CI: 0.79 to 0.92; p<0.001)	2.2 years
Long-Term Intervention with Pravastatin in Ischaemic Disease (LIPID)	Stable CAD, prior MI<br>n = 9014	Pravastatin 40mg<br>vs. placebo	3 months – 3 years	Mortality from CHD	RRR 24%<br>(95% CI: 12% to 35%; p<0.001)	6.1 years
ODYSSEY OUTCOMES	Stable CAD, prior MI<br>n = 18924	Alirocumab 75mg<br>vs. placebo	2.6 months (1.7 – 4.4)<br>Median (IQR)	MACE (death from CHD, nonfatal MI, fatal or nonfatal ischemic stroke, UAP requiring hospitalization)	HR 0.85<br>(95% CI: 0.78 to 0.93; p<0.001)	2.8 years

Supplementary table 1 Landmark trials on the effect of LDL-C lowering therapy on cardiovascular endpoints. Table is organized by study population from no CAD – stable CAD, prior MI. Abbreviations: CAD = coronary artery disease, FU = follow-up, HR = hazard ratio, IQR = interquartile range, MI = myocardial infarction, MACE = major adverse cardiac events, RR = relative risk, RRR = relative risk reduction, SCD = sudden cardiac death, SD = standard deviation, UAP = unstable angina pectoris
